# Effect of β-blockers and calcium channel blockers on shock index predictability in patients suffering from urosepsis

**DOI:** 10.1186/cc12163

**Published:** 2013-03-19

**Authors:** J Cliche, J St-Cyr Bourque, R Daoust, J Chauny, J Paquet, E Piette

**Affiliations:** 1 Sacré-Coeur Hospital, Montreal, Canada

## Introduction

In our busy emergency departments the identification of patients at risk of rapid hemodynamic decompensation is crucial. The shock index (SI) (pulse/systolic blood pressure) is a non-invasive clinical sign associated with more complications if higher than 0.7. The effect of β-blockers (BB) and calcium channel blockers (CCB) on the SI has not yet been described. Most studies excluded these patients, owing to the medications' effect on the cardiac pulse. Considering that BB and CCB are commonly prescribed, we studied their effect on the SI's predictability on 30-day mortality in a urosepsis population.

## Methods

This single-center *post-hoc *analysis of prospectively collected data was conducted in an academic Canadian emergency department (ED) between March 2008 and February 2011. Data were extracted from two institutional databases. We included patients with a final diagnosis of urosepsis, sepsis, pyelonephritis and urinary tract infection. Selected patients also had a documented positive urine culture. The SI predictability on 30-day mortality was calculated for patients taking BB and/or CCB as well as patients taking none of these medications. Sensitivity and specificity were determined using ROC curves. *t *tests were used to compare mean SI between both groups.

## Results

Our urosepsis population contained 364 patients, of which 129 (35.4%) were either using a BB, a CCB or both before their admission. Mean age was 74.9 years and 48.1% of the patients were women. A total of 36 patients died (9.9%) in a 30-day period. The group taking either BB or CCB had a significantly lower mean SI (0.76, 95% CI: 0.72 to 0.81 vs. 0.93, 95% CI: 0.89 to 0.98, *P <*0.0001). In our urosepsis population, a SI of 0.7 had a sensitivity of 0.76 and a specificity of 0.27 (area under the curve (AUC): 0.596) for patients taking neither BB nor CCB. In the group taking either or both medications, a SI of 0.7 had a sensitivity of 0.57 and a specificity of 0.42 (AUC: 0.578). In both groups, lowering the SI to 0.5 increased the sensitivity to more than 0.95 but lowered specificity significantly.

## Conclusion

To our knowledge this is the only study analysing the effect of BB and CCB on the SI predictability of 30-day mortality. Our results indicate that SI cannot be used to accurately predict mortality with patients suffering from urosepsis. In both our groups, SI performance was poor, as shown by the ROC curves. BB or CCB did not influence these results.

